# Supplementary material for: Type II innate lymphoid cell plasticity contributes to impaired reconstitution after allogeneic hematopoietic stem cell transplantation
Source: Nat Commun. 2024 Jul 17;15:6000. doi: 10.1038/s41467-024-50263-7 (PMC11255294; doi:10.1038/s41467-024-50263-7)
Supplement: Supplementary file 1 — Supplementary Information [file 41467_2024_50263_MOESM1_ESM.pdf]

Type II Innate Lymphoid Cell Plasticity Contributes to Impaired Reconstitution After Allogeneic Hematopoietic Stem Cell Transplantation

Sonia J. Laurie[1±], Joseph P. Foster II[1, 2±], Danny W. Bruce[1], Hemamalini Bommiasamy[1], Oleg V. Kolupaev[1, 10], Mostafa Yazdimamaghani[1], Samantha G. Pattenden[3], Nelson J. Chao[4], Stefanie Sarantopoulos[4], Joel S. Parker[1, 6], Ian J. Davis[1, 2, 5, 6], and Jonathan S. Serody[1, 7, 8, 9]

[1] Lineberger Comprehensive Cancer Center, University of North Carolina School of Medicine, Chapel Hill, NC, 27599, USA.

[2] Curriculum in Bioinformatics & Computational Biology, University of North Carolina, Chapel Hill, NC, 27599, USA.

[3] Center for Integrative Chemical Biology and Drug Discovery, Division of Chemical Biology and Medicinal Chemistry, University of North Carolina Eshelman School of Pharmacy, Chapel Hill, NC, 27599, USA.

[4] Division of Hematologic Malignancies and Cellular Therapy, Department of Medicine, Duke University Medical Center, Duke Cancer Institute, Durham, NC, 27701, USA.

[5] Division of Pediatric Hematology-Oncology, Department of Pediatrics, University of North Carolina School of Medicine, Chapel Hill, NC, 27599, USA.

[6] Department of Genetics, University of North Carolina, Chapel Hill, NC, 27599, USA.

[7] Department of Microbiology & Immunology, University of North Carolina School of Medicine, Chapel Hill, NC, 27599, USA.

[8] Division of Hematology, University of North Carolina School of Medicine, Chapel Hill, NC, 27599, USA.

[9] Department of Medicine, University of North Carolina School of Medicine, Chapel Hill, NC, 27599, USA.

[10] Present address: Duke Eye Center, Duke University, Durham, NC, 27701

This PDF file contains the following Supplementary Information:

Supplementary Figures 1-5

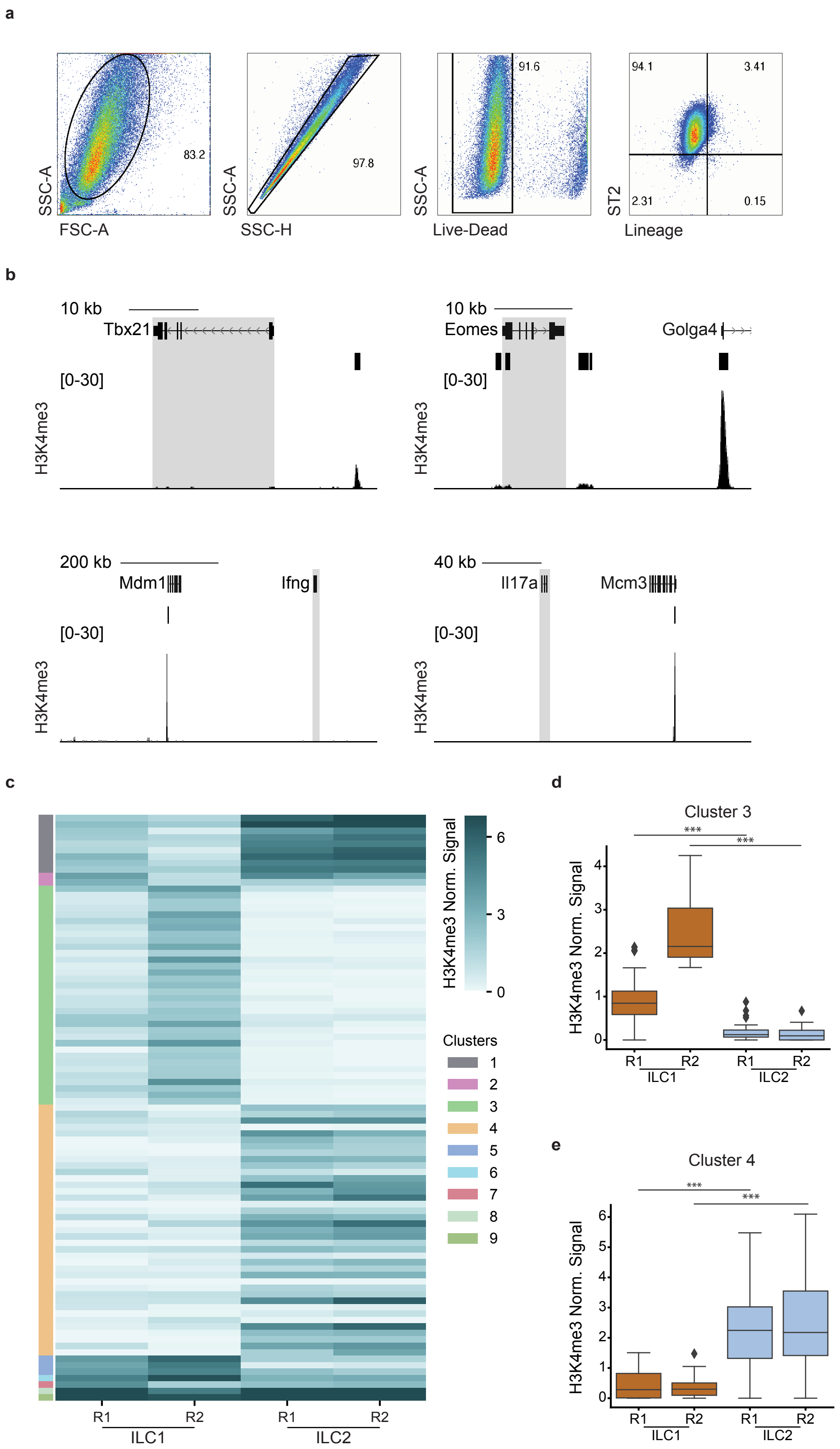
a
SSC-A
FSC-A
83.2
SSC-A
SSC-H
97.8
SSC-A
Live-Dead
91.6
ST2
Lineage
94.1
3.41
2.31
0.15
b
10 kb
Tbx21
[0-30]
H3K4me3
10 kb
Eomes
Golga4
[0-30]
H3K4me3
200 kb
Mdm1
Ifng
[0-30]
H3K4me3
40 kb
Il17a
Mcm3
[0-30]
H3K4me3
c
H3K4me3 Norm. Signal
6
3
0
Clusters
1
2
3
4
5
6
7
8
9
R1
R2
ILC1
R1
R2
ILC2
d
Cluster 3
H3K4me3 Norm. Signal
***
***
R1
R2
ILC1
R1
R2
ILC2
e
Cluster 4
H3K4me3 Norm. Signal
***
***
R1
R2
ILC1
R1
R2
ILC2

Supplementary Figure 1: Characterizing the chromatin landscape of ex vivo expanded ILC2s. **(a)** Flow cytometry gating scheme used to define ILC2s from the peritoneum and mesenteric lymph nodes as presented in Fig. 1e. **(b)** Representative tracks of H3K4me3 signal at ILC1 lineage defining genes *Tbx21, Ifng,* and *Eomes*, and ILC3 hallmark gene *Il17a*. Tick marks above tracks indicate peak calls by MACS2. **(c)** Differential H3K4me3 signal from -300 bp to +500 bp around all TSSs was identified using DeSeq2 (Likelihood ratio test, p adj < 0.05). These TSSs were then grouped by hierarchical clustering. Heatmap shows normalized H3K4me3 signal at these differential regions for both ILC1 and ILC2 cells. R1 and R2 indicate replicate 1 and 2 of the experiment. **(d, e)** Bar plots depicting the distribution of H3K4me3 signal from -300 bp to +500 bp around TSSs in cluster 3. R1 and R2 indicate replicate 1 and 2 of the experiment. (**d**) and cluster 4 (**e**). Significance was determined using Mann Whitney U Test. *** indicates $p < 0.001$. Box plot lines represent min/max values.

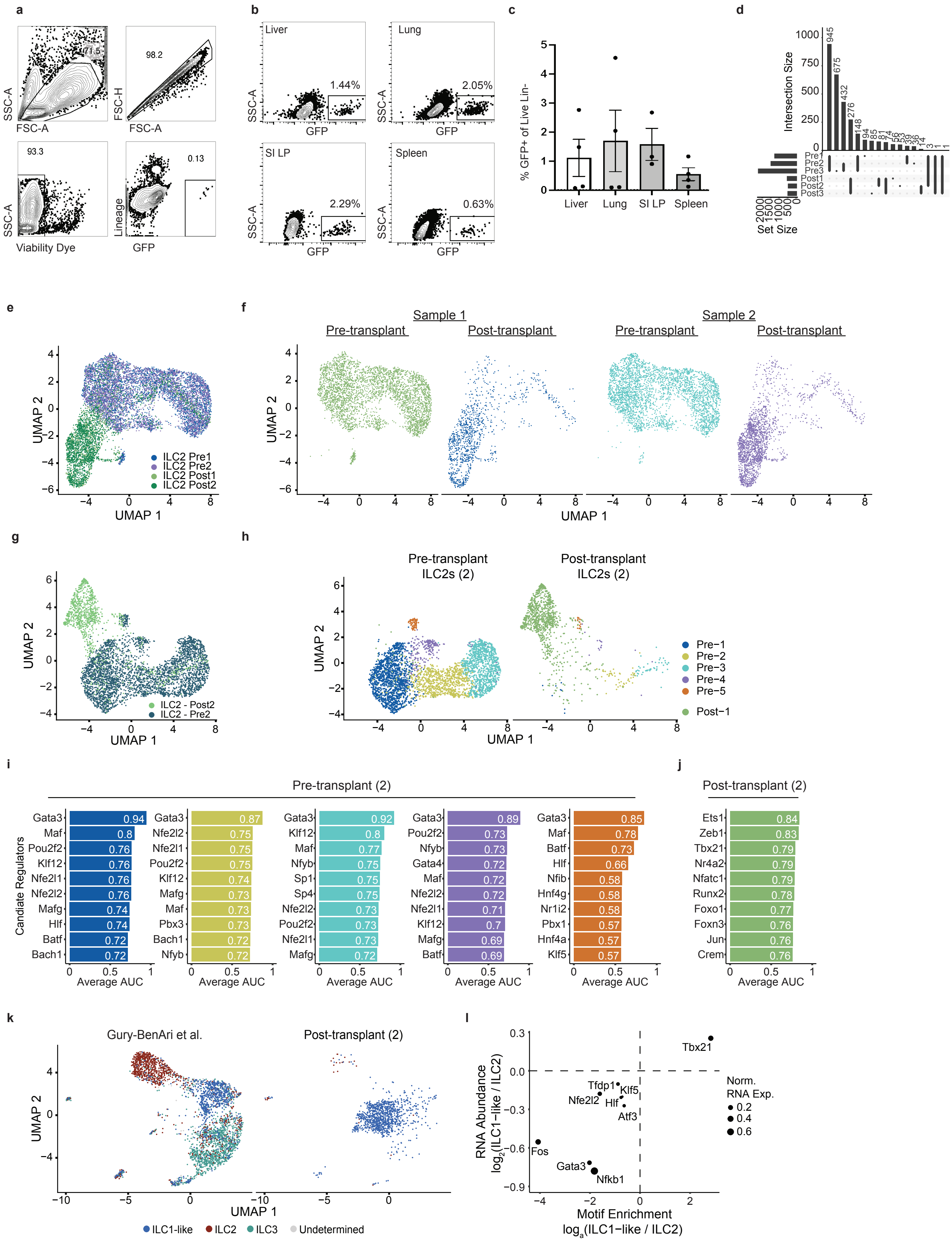
a
71.5
SSC-A
FSC-A
98.2
FSC-H
FSC-A
93.3
SSC-A
Viability Dye
0.13
Lineage
GFP
b
Liver
1.44%
SSC-A
GFP
Lung
2.05%
SSC-A
GFP
SI LP
2.29%
SSC-A
GFP
Spleen
0.63%
SSC-A
GFP
c
% GFP+ of Live Lin-
Liver
Lung
SI LP
Spleen
d
Intersection Size
945
675
432
276
148
94
85
81
74
56
53
39
36
14
3
1
1
Pre1
Pre2
Pre3
Post1
Post2
Post3
Set Size
e
UMAP 2
UMAP 1
ILC2 Pre1
ILC2 Pre2
ILC2 Post1
ILC2 Post2
f
Sample 1
Pre-transplant
Post-transplant
Sample 2
Pre-transplant
Post-transplant
UMAP 2
UMAP 1
g
UMAP 2
UMAP 1
ILC2 - Post2
ILC2 - Pre2
h
Pre-transplant
ILC2s (2)
Post-transplant
ILC2s (2)
UMAP 2
UMAP 1
Pre−1
Pre−2
Pre−3
Pre−4
Pre−5
Post−1
i
Pre-transplant (2)
Candidate Regulators
Gata3 0.94
Maf 0.8
Pou2f2 0.76
Klf12 0.76
Nfe2l1 0.76
Nfe2l2 0.76
Mafg 0.74
Hlf 0.74
Batf 0.72
Bach1 0.72
Gata3 0.87
Nfe2l2 0.75
Nfe2l1 0.75
Pou2f2 0.75
Klf12 0.74
Mafg 0.73
Maf 0.73
Pbx3 0.73
Bach1 0.72
Nfyb 0.72
Gata3 0.92
Klf12 0.8
Maf 0.77
Nfyb 0.75
Sp1 0.75
Sp4 0.75
Nfe2l2 0.73
Pou2f2 0.73
Nfe2l1 0.73
Mafg 0.72
Gata3 0.89
Pou2f2 0.73
Nfyb 0.73
Gata4 0.72
Maf 0.72
Nfe2l2 0.72
Nfe2l1 0.71
Klf12 0.7
Mafg 0.69
Batf 0.69
Gata3 0.85
Maf 0.78
Batf 0.73
Hlf 0.66
Nfib 0.58
Hnf4g 0.58
Nr1i2 0.58
Pbx1 0.57
Hnf4a 0.57
Klf5 0.57
Average AUC
j
Post-transplant (2)
Ets1 0.84
Zeb1 0.83
Tbx21 0.79
Nr4a2 0.79
Nfatc1 0.79
Runx2 0.78
Foxo1 0.77
Foxn3 0.76
Jun 0.76
Crem 0.76
Average AUC
k
Gury-BenAri et al.
Post-transplant (2)
UMAP 2
UMAP 1
ILC1-like
ILC2
ILC3
Undetermined
l
RNA Abundance
log2(ILC1−like / ILC2)
Tbx21
Tfdp1
Klf5
Nfe2l2
Hlf
Atf3
Fos
Gata3
Nfkb1
Norm.
RNA Exp.
0.2
0.4
0.6
Motif Enrichment
loga(ILC1−like / ILC2)

Supplementary Figure 2: A second sample confirms the emergence of ILC1-like exILC2s after murine allogeneic hematopoietic stem cell transplantation. (a) Flow cytometric gating strategy for identifying lineage-negative, GFP$^+$ cells in aGVHD target organs (liver, lung, small intestine) and the spleen following allo-HSCT. **(b)** Representative flow plots indicating the accumulation of lineage-negative, GFP$^+$ cells in selected aGVHD target organs (liver, lung, small intestine) and the spleen 14 to 20 days after allo-HSCT. **(c)** Quantification by flow cytometry of frequency lineage-GFP$^+$ cells across aGVHD target organs after allo-HSCT. Results represent 3 independent experiments with a total n of 3-4 per group. Error bars in **c** represent SEM. **(d)** Upset plot depicting number of genes shared between all combinations of pre- and post-transplant cluster gene sets. **(e, f)** UMAP plot showing integration of pre-transplant per-nucleus RNA (2 replicates) abundance and post-transplant per-nucleus RNA (2 replicates) (**e**). Data are split by whether cells are pre- or post-transplant (**f**). **(g, h)** UMAP representation of second replicate of pre-transplant (**g**, blue) and post-transplant (**g**, green) ILC2s. (**h**) Cells were clustered on normalized gene expression. **(i, j)** Bar plots showing AUC statistic for each of the putative transcription factors for each of the pre-transplant (**i**) and post-transplant clusters (**j**). **(k)** UMAP representation of the second replicate of integrated post-transplant ILC2s and CD127$^+$ cells isolated from the small intestine of healthy mice in a previous study[1]. UMAP depicts a low-dimensional representation of integrated batch corrected RNA abundance estimates. Cells were classified by calculating the average rank of genes associated with each type of ILC and labeling each cell with the cell type with the highest rank. Unknown signifies a tie in ranking. **(l)** Putative regulators of ILC1-like (Supplementary Fig. 2k, second replicate) and ILC2s (Supplementary Fig. 2k) were identified as transcription factor genes with differential RNA abundance and motifs accessibilities (Wilcox rank sum test, RNA.padj < 0.05, RNA.logFC > 0 and motif.padj < 0.05, motif.logFC > 0). Scatter plot shows the relationship between changes in RNA abundance and changes in motif enrichment. Size of dot indicates average $\log_2$ normalized gene expression.

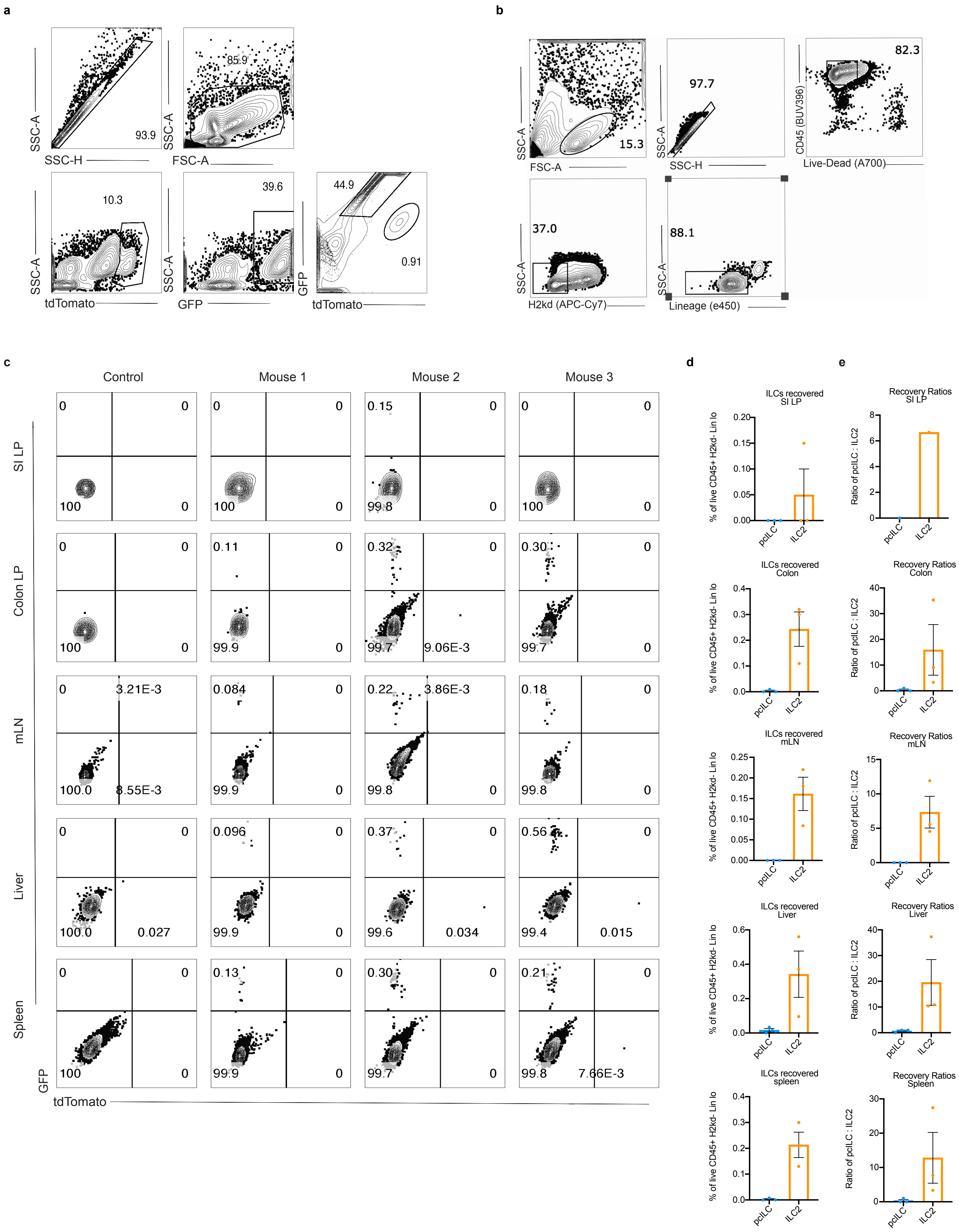

a
SSC-A
SSC-H
93.9
SSC-A
FSC-A
85.9
SSC-A
tdTomato
10.3
SSC-A
GFP
39.6
GFP
tdTomato
44.9
0.91
b
SSC-A
FSC-A
15.3
SSC-A
SSC-H
97.7
CD45 (BUV396)
Live-Dead (A700)
82.3
SSC-A
H2kd (APC-Cy7)
37.0
SSC-A
Lineage (e450)
88.1
c
Control
Mouse 1
Mouse 2
Mouse 3
SI LP
Colon LP
mLN
Liver
Spleen
GFP
tdTomato
0
100
0.15
99.8
0.11
99.9
0.32
99.7
9.06E-3
0.30
3.21E-3
100.0
8.55E-3
0.084
0.22
3.86E-3
0.18
0.096
0.027
0.37
99.6
0.034
0.56
99.4
0.015
0.13
0.21
7.66E-3
d
ILCs recovered SI LP
ILCs recovered Colon
ILCs recovered mLN
ILCs recovered Liver
ILCs recovered spleen
% of live CD45+ H2kd- Lin lo
pcILC
ILC2
e
Recovery Ratios SI LP
Recovery Ratios Colon
Recovery Ratios mLN
Recovery Ratios Liver
Recovery Ratios Spleen
Ratio of pcILC : ILC2

Supplementary Figure 3: The conversion of ILC2s to an ILC1-like fate after HSCT is not a result of out-competition by a small founder population of ILC1 cells. **a**, Lethally irradiated B6D2 mice received allogeneic (C57BL/6) T cell depleted bone marrow along with 50 tdTomato$^+$ ILC1-like pcILC2 cells for each eGFP$^+$ ILC2 cell infused, along with an equal number of T cells for a total of $4x10^6$ total ILCs and T cells each. **b**, 14 days after transplantation animals were sacrificed and the liver, mesenteric lymph nodes, and lamina propria of the colon and small intestine were harvested and tissue-resident ILCs were quantified by flow cytometry as the frequency of live cells. **c**, To specifically identify pcILCs or ILC2s fluorescent genetic tags, the expression of tdTomato and eGFP, respectively, were assessed as (**d**) a percentage or (**e**) ratios. Error bars in **d** and **e** represent SEM.

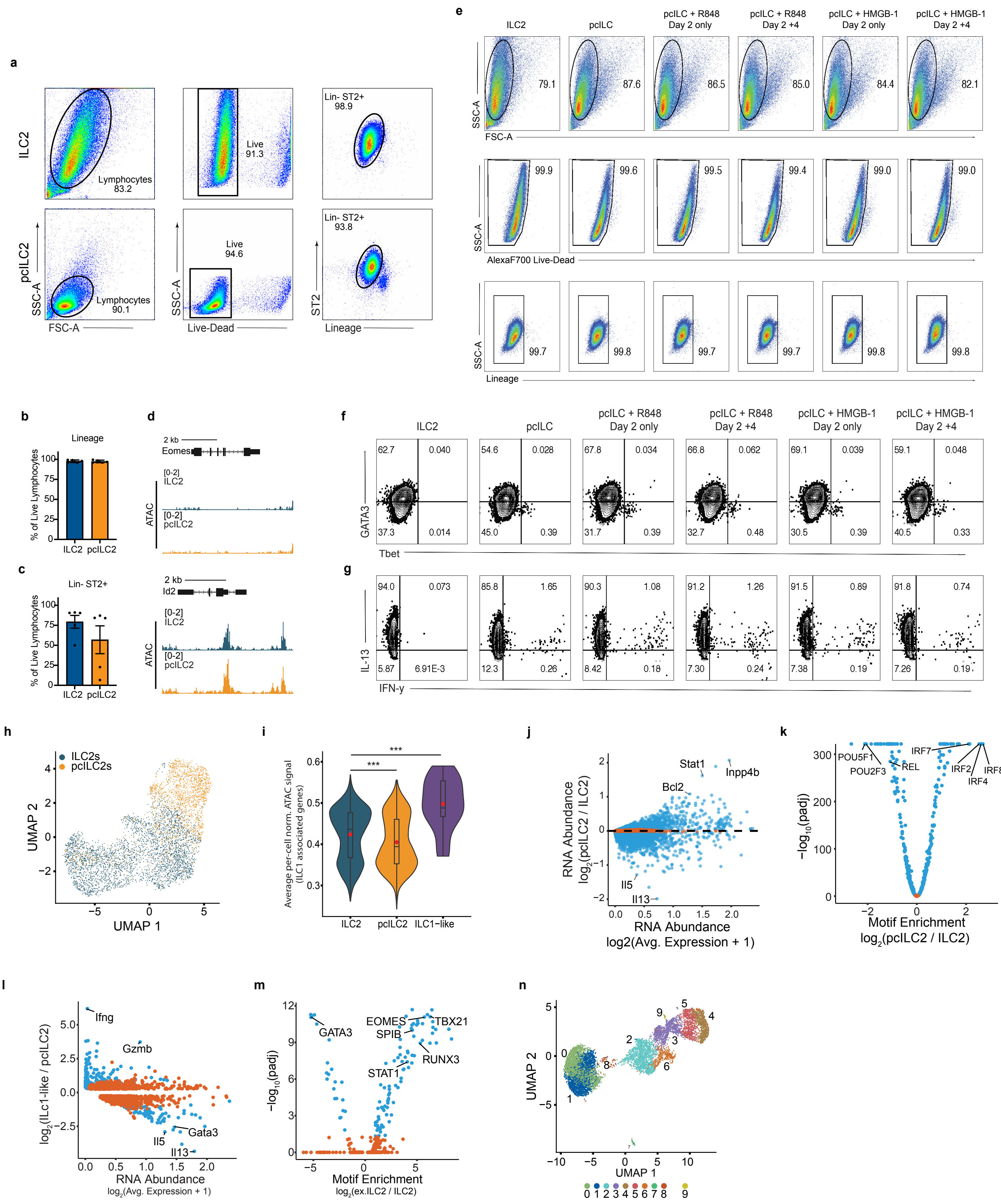
a
ILC2
pcILC2
Lymphocytes 83.2
Live 91.3
Lin- ST2+ 98.9
Lymphocytes 90.1
Live 94.6
Lin- ST2+ 93.8
SSC-A
FSC-A
Live-Dead
ST2
Lineage
e
ILC2
pcILC
pcILC + R848 Day 2 only
pcILC + R848 Day 2 +4
pcILC + HMGB-1 Day 2 only
pcILC + HMGB-1 Day 2 +4
79.1 87.6 86.5 85.0 84.4 82.1
99.9 99.6 99.5 99.4 99.0 99.0
99.7 99.8 99.7 99.7 99.8 99.8
AlexaF700 Live-Dead
b
Lineage
% of Live Lymphocytes
c
Lin- ST2+
d
2 kb
Eomes
Id2
[0-2] ILC2
[0-2] pcILC2
ATAC
f
GATA3
Tbet
62.7 0.040 37.3 0.014
54.6 0.028 45.0 0.39
67.8 0.034 31.7 0.39
66.8 0.062 32.7 0.48
69.1 0.039 30.5 0.39
59.1 0.048 40.5 0.33
g
IL-13
IFN-y
94.0 0.073 5.87 6.91E-3
85.8 1.65 12.3 0.26
90.3 1.08 8.42 0.18
91.2 1.26 7.30 0.24
91.5 0.89 7.38 0.19
91.8 0.74 7.26 0.19
h
ILC2s
pcILC2s
UMAP 1
UMAP 2
i
Average per-cell norm. ATAC signal (ILC1 associated genes)
ILC2 pcILC2 ILC1−like
***
j
Stat1
Inpp4b
Bcl2
Il5
Il13
RNA Abundance log2(Avg. Expression + 1)
RNA Abundance log2(pcILC2 / ILC2)
k
POU5F1
POU2F3
REL
IRF7
IRF2
IRF8
IRF4
−log10(padj)
Motif Enrichment log2(pcILC2 / ILC2)
l
Ifng
Gzmb
Gata3
Il5
Il13
log2(ILc1-like / pcILC2)
RNA Abundance log2(Avg. Expression + 1)
m
GATA3
EOMES
SPIB
TBX21
RUNX3
STAT1
−log10(padj)
Motif Enrichment log2(ex.ILC2 / ILC2)
n
UMAP 1
UMAP 2
0 1 2 3 4 5 6 7 8 9

Supplementary Figure 4: (a) Flow cytometry gating scheme used to define ILC2s and pcILC2s after 6 days of in vitro expansion, as presented in Fig. 3b and 3c. **(b-c)** Summary of expression of Lineage proteins and the IL-33R ST2 on ILC2s and pcILC2s after culture. (**d**) Representative tracks of normalized ATAC signal at ILC1-associated Eomes and ILC-associated Id2. **(e)** Flow cytometric assessment of viable, blasting, lineage negative ILC2s and pcILC2s grow in the presence or absence of resiquimod (10 µg/ml) or HMGB-1 (50 ng/ml). (**f**) Quantitation of expression of TFs Tbet and GATA3 and (**g**) secretion of IL-13 and IFN-y following 4 hours of in vitro stimulation with Cell Stimulation cocktail and intracellular staining. **(h)** UMAP plot showing 5,188 nuclei. Each point is annotated based on cytokine treatment. **(h, i)** The average chromatin accessibility at genes previously identified as specifically expressed in ILC1[1]. The ILC2 population includes clusters 2 – 7 (blue), pcILC2 includes clusters 0, 1 and 8 (orange), and ILC1-like cells include cluster 9 (purple) from Supplementary Fig. 3g. Mean value is shown (red dot). Box shows interquartile range. **(k)** MA plot showing $\log_2$ fold change and average RNA abundance of genes differentially expressed between ILC2 and pcILC2 (two-sided Wilcoxon rank sum test). Blue dots indicate genes with p adj < 0.05. **(l)** Volcano plot depicting motifs enriched in either pcILC2 or ILC2s. Blue dots indicate genes with p adj < 0.05 (two-sided Wilcoxon rank sum test). **(j)** UMAP representation of pcILC2 and ILC2s integrated using transcriptome data. Points (each cell) were clustered on normalized RNA abundance signal. **(m)** MA plot showing $\log_2$ fold change and average RNA abundance of genes differentially expressed between ILC1-like cells (cluster 9 Supplementary Fig. 3g) and ILC2s. Blue dots indicate genes with p adj < 0.05. **(n)** Volcano plot depicting motifs enriched in either ILC1-like cells (cluster 9 Supplementary Fig. 3g) or ILC2s. Blue dots indicate genes with p adj < 0.05.

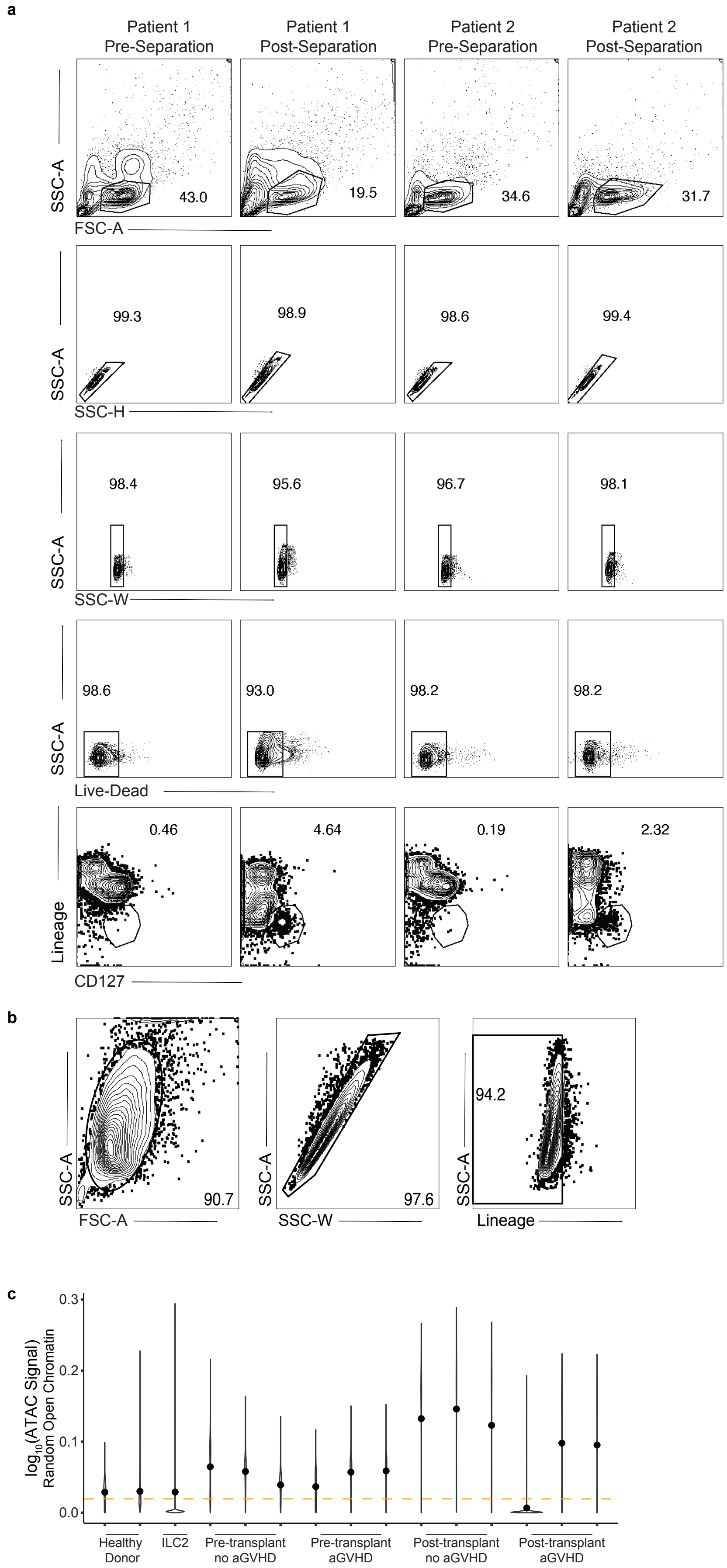
a
Patient 1 Pre-Separation
Patient 1 Post-Separation
Patient 2 Pre-Separation
Patient 2 Post-Separation
SSC-A
FSC-A
43.0
19.5
34.6
31.7
SSC-H
99.3
98.9
98.6
99.4
SSC-W
98.4
95.6
96.7
98.1
Live-Dead
98.6
93.0
98.2
98.2
Lineage
CD127
0.46
4.64
0.19
2.32
b
90.7
97.6
94.2
c
$\log_{10}$(ATAC Signal)
Random Open Chromatin
0.3
0.2
0.1
0.0
Healthy Donor
ILC2
Pre-transplant no aGVHD
Pre-transplant aGVHD
Post-transplant no aGVHD
Post-transplant aGVHD

Supplementary Figure 5: Average normalized ATAC signal at hILC2-associated sites of chromatin accessibility per nucleus.

(a) Flow cytometric analysis of healthy donor human PB pre- and post- enrichment with the STEMCELL Technologies RosetteSep human ILC2 enrichment kit looking at the frequency of lymphocytes (row 1), singlets (rows 2-3), live (row 4), lineage- CD127+ ILCs (row 5). Following enrichment, cells were expanded in culture for 14-21 days. **(b)** Representative flow cytometry plots depicting the gating strategy to identify highly pure lineage negative cells in advance of further flow cytometric or sequencing readouts as in Figure 4b-d. **(c)** Average normalized ATAC signal at hILC2-associated sites of CAs as defined in Fig. 4b was calculated per nucleus for each patient sample. Violin plots show the distribution of this per-nucleus signal. Orange dotted line indicates 10th percentile of signal. Black dots indicate mean of each distribution.

References

1. Gury-BenAri, M. *et al.* The Spectrum and Regulatory Landscape of Intestinal Innate Lymphoid Cells Are Shaped by the Microbiome. *Cell* **166**, 1231-1246 e13 (2016).
