# Supplementary material for: Type II innate lymphoid cell plasticity contributes to impaired reconstitution after allogeneic hematopoietic stem cell transplantation
Source: Nat Commun. 2024 Jul 17;15:6000. doi: 10.1038/s41467-024-50263-7 (PMC11255294; doi:10.1038/s41467-024-50263-7)
Supplement: Supplementary file 3 — Description of Additional Supplementary Files [file 41467_2024_50263_MOESM3_ESM.pdf]

Description of Additional Supplementary Files

Supplementary Data 1. Characteristics of six adult allo-HSCT recipients, half of whom were diagnosed with aGVHD in the first 100 days post-transplant, and half of whom did not develop aGvHD during this time-period. Host-derived PBMCs were collected prior to transplantation and donor-derived PBMCs were collected post-transplantation at the time of aGVHD diagnosis (approximately 4-6 weeks), or three months post-transplant in stable recipients.

Supplementary Data 2. Table including the format, fluorochrome conjugation (if applicable), target species and antigen, along with clone information, catalog and lot numbers and antibody dilutions for all applications presented herein.

Supplementary Data 3. Sequences for custom Nextera PCR primers used for for ATAC-seq amplification as described in Methods.
