# Supplementary material for: Type II innate lymphoid cell plasticity contributes to impaired reconstitution after allogeneic hematopoietic stem cell transplantation
Source: Nat Commun. 2024 Jul 17;15:6000. doi: 10.1038/s41467-024-50263-7 (PMC11255294; doi:10.1038/s41467-024-50263-7)
Supplement: Supplementary file 7 — Reporting Summary [file 41467_2024_50263_MOESM7_ESM.pdf]

nature portfolio

Corresponding author(s): Jonathan S. Serody, MD

Last updated by author(s): 05/30/2024

Reporting Summary

Nature Portfolio wishes to improve the reproducibility of the work that we publish. This form provides structure for consistency and transparency in reporting. For further information on Nature Portfolio policies, see our Editorial Policies and the Editorial Policy Checklist.

Statistics

For all statistical analyses, confirm that the following items are present in the figure legend, table legend, main text, or Methods section.

n/a	Confirmed	
☐	☒	The exact sample size (*n*) for each experimental group/condition, given as a discrete number and unit of measurement
☐	☒	A statement on whether measurements were taken from distinct samples or whether the same sample was measured repeatedly
☐	☒	The statistical test(s) used AND whether they are one- or two-sided<br>*Only common tests should be described solely by name; describe more complex techniques in the Methods section.*
☐	☒	A description of all covariates tested
☐	☒	A description of any assumptions or corrections, such as tests of normality and adjustment for multiple comparisons
☐	☒	A full description of the statistical parameters including central tendency (e.g. means) or other basic estimates (e.g. regression coefficient) AND variation (e.g. standard deviation) or associated estimates of uncertainty (e.g. confidence intervals)
☐	☒	For null hypothesis testing, the test statistic (e.g. *F*, *t*, *r*) with confidence intervals, effect sizes, degrees of freedom and *P* value noted<br>*Give P values as exact values whenever suitable.*
☒	☐	For Bayesian analysis, information on the choice of priors and Markov chain Monte Carlo settings
☐	☒	For hierarchical and complex designs, identification of the appropriate level for tests and full reporting of outcomes
☐	☒	Estimates of effect sizes (e.g. Cohen's *d*, Pearson's *r*), indicating how they were calculated

Our web collection on statistics for biologists contains articles on many of the points above.

Software and code

Policy information about availability of computer code

Data collection	Previously published data used in manuscript:<br>H3K4me3 ChIP-seq fastq files from Gury-BenAri and Thaiss et al. 2016 Cell study were downloaded from GEO (GSE85156)<br>RNA-seq fastq files from Bruce et al. was downloaded from GEO (GSE95811).<br>MARS-seq UMI Count generated by Gury-BenAri and Thaiss et al. was downloaded from GEO (GSE85152).
Data analysis	The code used to perform analyses and generate figures for manuscript is available via GitHub (https://github.com/j-foster2/GvHD). As suggested by the reviewer and to facilitate easier reproduction of figures, these scripts were run within an Apptainer container.<br><br>The Apptainer definition file has also been uploaded to the GitHub repository and can be used to produce the container. Additionally, walkthroughs on how the code was run to generate the figures are included in the GitHub README file. These scripts take count matrices and bam files as input. The pipelines used to generate these files are described in the methods section of the manuscript and the GitHub README file.<br><br>ChIP-seq<br>star (v. 2.5.2b)<br>samtools (v. 1.3.1)<br>bedtools (v. 2.26)<br>fastqc (v. 0.11.5)<br>fastx_toolkit (v. 0.0.14)<br>cutadapt (v. 1.12)<br>deepTools (v.3.5.1)

MACS2 (v.2.1.2)
DEseq2 (v. 1.40.2)

RNA-seq
star (v. 2.5.2b)
samtools (v. 1.3.1)
bedtools (v. 2.26)
fastqc (v. 0.11.5)
fastx_toolkit (v. 0.0.14)
cutadapt (v. 1.12)
deepTools (v.3.5.2)
Salmon (v0.11.3)

ATAC-seq
star (v. 2.5.2b)
samtools (v. 1.3.1)
bedtools (v. 2.26)
fastqc (v. 0.11.5)
fastx_toolkit (v. 0.0.14)
cutadapt (v. 1.12)
MACS (v. 2.1.2)
DESeq2 (v. 1.40.2)
HOMER (v. 4.11.1)

single nucleus RNA and ATAC-seq (Multiome)
cellranger-arc count (v. 2.0.0)
Seurat (v. 4.3.0.1)
Signac (v. 1.10.0)
chromVar (v. 1.22.1)

scATAC-seq
cellranger-atac count (v. 2.0.0)
Seurat (v. 4.3.0.1)
Signac (v. 1.10.0)
g:profiler (v. 0.2.2)

For manuscripts utilizing custom algorithms or software that are central to the research but not yet described in published literature, software must be made available to editors and reviewers. We strongly encourage code deposition in a community repository (e.g. GitHub). See the Nature Portfolio guidelines for submitting code & software for further information.

Data

Policy information about availability of data

All manuscripts must include a data availability statement. This statement should provide the following information, where applicable:

- Accession codes, unique identifiers, or web links for publicly available datasets
- A description of any restrictions on data availability
- For clinical datasets or third party data, please ensure that the statement adheres to our policy

Sequence data that support the findings of this study have been deposited in GEO with accession codes as follows:

To review GEO accession GSE231999:
Go to https://www.ncbi.nlm.nih.gov/geo/query/acc.cgi?acc=GSE231999
Enter token irwtqwauzneldwv into the box

To review GEO accession GSE232000:
Go to https://www.ncbi.nlm.nih.gov/geo/query/acc.cgi?acc=GSE232000
Enter token azohewighfofbqt into the box

To review GEO accession GSE232001:
Go to https://www.ncbi.nlm.nih.gov/geo/query/acc.cgi?acc=GSE232001
Enter token ihafwqecrvgjdgb into the box

To review GEO accession GSE232002:
Go to https://www.ncbi.nlm.nih.gov/geo/query/acc.cgi?acc=GSE232002
Enter token clytoqgmbdsndsx into the box

To review GEO accession GSE232003:
Go to https://www.ncbi.nlm.nih.gov/geo/query/acc.cgi?acc=GSE232003
Enter token kpeliamsltorfof into the box

Additionally, we have deposited relevant information into a GitHub repository which contains an introduction summarizing the purpose of therepository and its connection to the associated research paper. The GitHub can be accessed at https://github.com/j-foster2/GvHD/releases/tag/v1.0.1. The code has also been archived in the Zenodo repository (https://doi.org/10.5281/zenodo.11396929).

Research involving human participants, their data, or biological material

Policy information about studies with human participants or human data. See also policy information about sex, gender (identity/presentation), and sexual orientation and race, ethnicity and racism.

Reporting on sex and gender	Sex and/or gender were not considered in the study design as specimen selection was limited to a small pool of experimentally eligible samples. No sex or gender analyses were carried out.
Reporting on race, ethnicity, or other socially relevant groupings	Due to lack of PHI disclosure to organizers of this particular study, no decisions were made regarding exclusion or exclusion of samples based on race, ethnicity, or other socially relevant groupings.
Population characteristics	Peripheral blood samples were collected from 12 adults who underwent HSCT at the Duke Adult Bone Marrow Transplant Clinic in Durham, NC between January of 2015 and April of 2017. The average patient age at the time of HSCT was 56 years, and all included patients were White non-hispanic, with a transplant indication of myelodysplastic syndrome. All patients received calcineurin inhibition and short course methotrexate for GvHD prophylaxis.
Recruitment	Adults receiveing allogeneic hematopoietic stem cell transplantations consented to have blood-derived PBMCs biobanked under Duke University IRB study protocol Pro00110250. We requested samples from 3-6 patients after alloHSCT with a diagnosis of acute graft-versus-host disease and 3-6 that were stable. Additionally, we requested a pre-HSCT sample and then one drawn as close as possible to the time of the aGVHD diagnosis, as well as 3 months and 1 year where available.
Ethics oversight	As described above, patient samples were obtained under Duke University IRB study protocol Pro00110250, PI: Dr. Nelson J. Chao.

Note that full information on the approval of the study protocol must also be provided in the manuscript.

Field-specific reporting

Please select the one below that is the best fit for your research. If you are not sure, read the appropriate sections before making your selection.

☒ Life sciences ☐ Behavioural & social sciences ☐ Ecological, evolutionary & environmental sciences

For a reference copy of the document with all sections, see nature.com/documents/nr-reporting-summary-flat.pdf

Life sciences study design

All studies must disclose on these points even when the disclosure is negative.

Sample size	For GvHD experiments, sample sizes were chosen for the effect size needed based on our previous experience of the number of samples needed to demonstrate a significant difference in GvHD scoring between control and treated groups. For the scoring evaluation experiments, the inclusion of 9-12 recipients provided a power of 90% to detect a difference of 14 days in the median GvHD score of > 5 with an alpha error of < 0.05 between control and treated groups. For human experiments (Figure 4), sample size was limited by the number of adults transplanted at the Duke aBMT clinic who could have the time of their sample draws matched to fit the needs of our experimental design.
Data exclusions	Single Cell: We excluded nuclei with a total RNA read count > 25,000, total RNA read count < 1,000, total ATAC read count > 70,000, total ATAC read count < 5,000, and mitochondrial counts > 20% Cells AAGGATTAGCTCATAA-1_2, AGTGGACAGCTATTAG-1_2 were excluded from downstream analysis.
Replication	For transplant experiments, each group contained a minimum of 6 mice per group and independent experiments were performed a minimum of two times. Biological and technical replicates were both considered wherever possible. For multiomic single cell analysis of ILC2s following transplantation, experiments where cell numbers were severely limited, we were able to prepare libraries and sequence samples in duplicate.
Randomization	For in vivo murine experiments, age-matched animals were randomly assigned into control or ILC2 recipient groups. For in vitro cell culture experiments, cells were randomly assigned into different treatment groups.
Blinding	For in vivo experiments we did not have a specific protocol to blind the investigators, however all animals underwent transplantation and cytokine treatment (respectively) at the same time, and blinding was not required for scoring as animals were euthanized prior to the development of fulminant graft versus host disease. For experiments involving human samples, investigators were not blinded to patient groups, however the preparation of single-cell ATAC libraries occurred in two batches, each of which contained the same number of samples from patients with and without aGVHD.

Reporting for specific materials, systems and methods

We require information from authors about some types of materials, experimental systems and methods used in many studies. Here, indicate whether each material, system or method listed is relevant to your study. If you are not sure if a list item applies to your research, read the appropriate section before selecting a response.

Materials & experimental systems

n/a	Involved in the study	
☐	☒	Antibodies
☒	☐	Eukaryotic cell lines
☒	☐	Palaeontology and archaeology
☐	☒	Animals and other organisms
☒	☐	Clinical data
☒	☐	Dual use research of concern
☒	☐	Plants

Methods

n/a	Involved in the study	
☐	☒	ChIP-seq
☐	☒	Flow cytometry
☒	☐	MRI-based neuroimaging

Antibodies

Antibodies used

1. Biotinylated anti-mouse CD8a, clone 53-6.7, lot 2274308, eBioscience, Catalog #13-0081-82, Dilution: 2 ul ab/10^7 cells, https://www.thermofisher.com/antibody/product/CD8a-Antibody-clone-53-6-7-Monoclonal/13-0081-82

2. Biotinylated anti-mouse CD4, clone RM4.4, lot 2162240, eBioscience, Catalog # 13-0043-82, Dilution: 2 ul ab/10^7 cells, https://www.thermofisher.com/antibody/product/CD4-Antibody-clone-RM4-4-Monoclonal/13-0043-82

3. Biotinylated anti-mouse CD3e, clone 145-2C11, lot 2396777, eBioscience, Catalog # 13-0031-82, Dilution: 2 ul ab/10^7 cells, https://www.thermofisher.com/antibody/product/CD3e-Antibody-clone-145-2C11-Monoclonal/13-0031-82

4. Biotinylated anti-mouse gdTCR, clone UC7-12DS, lot 2162231, eBioscience, Catalog # 13-5811-82, Dilution: 2 ul ab/10^7 cells, https://www.thermofisher.com/antibody/product/TCR-gamma-delta-Antibody-clone-UC7-13D5-Monoclonal/13-5811-82

5. Biotinylated anti-mouse TER119, clone TER-119, lot 2174196, eBioscience, Catalog #13-5921-82, Dilution: 2 ul ab/10^7 cells, https://www.thermofisher.com/antibody/product/TER-119-Antibody-clone-TER-119-Monoclonal/13-5921-82

6. Biotinylated anti-mouse B220, clone RA3-6B2, lot 2083488, eBioscience, Catalog #13-0452-82, Dilution: 2 ul ab/10^7 cells, https://www.thermofisher.com/antibody/product/CD45R-B220-Antibody-clone-RA3-6B2-Monoclonal/13-0452-82

7. Biotinylated anti-mouse CD11b, clone M1/70, lot 2151423, eBioscience, Catalog #13-0112-82, Dilution: 2 ul ab/10^7 cells, https://www.thermofisher.com/antibody/product/CD11b-Antibody-clone-M1-70-Monoclonal/13-0112-82

8. Biotinylated anti-mouse NK1.1, clone PK126, lot 2396752, Invitrogen, Catalog #13-5941-82, Dilution: 2 ul ab/10^7 cells, https://www.thermofisher.com/antibody/product/NK1-1-Antibody-clone-PK136-Monoclonal/13-5941-82

9. Biotinylated anti-mouse CD11c, clone N418, lot 2151424, Invitrogen, Catalog #13-0114-82, Dilution: 2 ul ab/10^7 cells, https://www.thermofisher.com/antibody/product/CD11c-Antibody-clone-N418-Monoclonal/13-0114-82

10. Biotinylated anti-mouse CD19, clone MB19-1, lot 2171824, eBioscience, Catalog #13-0191-82, Dilution: 2 ul ab/10^7 cells, https://www.thermofisher.com/antibody/product/CD19-Antibody-clone-MB19-1-Monoclonal/13-0191-82

11. Biotinylated anti-mouse Ly6G, clone 1A8, lot B297876, BioLegend, Catalog #127604, Dilution: 2 ul ab/10^7 cells, https://www.biolegend.com/en-us/products/biotin-anti-mouse-ly-6g-antibody-4772

12. Biotinylated anti-mouse CD49b, clone DX5, lot 2183518, Invitrogen, Catalog #13-5971-82, Dilution: 2 ul ab/10^7 cells, https://www.thermofisher.com/antibody/product/CD49b-Integrin-alpha-2-Antibody-clone-DX5-Monoclonal/13-5971-82

13. Anti-mouse Lineage cocktail, eFluor450 conjugated, clones CD3 (17A2), CD45R/B220 (RA3-6B2), CD11b (M1/70), TER-119 (TER-119), Ly-G6 (Gr-1, RB6-8C5), lot 2324728, eBiosciences, Catalog #88-7772-72, Dilution 1:10-1:50, https://www.thermofisher.com/antibody/product/Mouse-Hematopoietic-Lineage-Antibody-Cocktail/88-7772-72

14. Anti-mouse Lineage cocktail, FITC conjugated, clones CD3 (17A2), CD45R/B220 (RA3-6B2), CD11b (M1/70), TER-119 (TER-119), Ly-G6 (Gr-1, RB6-8C5), lot 2324728, eBiosciences, Catalog #22-7770-72, Dilution 1:10-1:50, https://www.thermofisher.com/antibody/product/Mouse-Hematopoietic-Lineage-Antibody-clone-17A2-RA3-6B2-M1-70-TER-119-RB6-8C5-Cocktail/22-7770-72

15. Anti-mouse ST2 (IL-33R), PerCP-eFluor710 conjugated, clone RMST2-2, lot unknown, Invitrogen, Catalog #46-9333-82, Dilution 1:200, https://www.thermofisher.com/antibody/product/IL-33R-ST2-Antibody-clone-RMST2-33-Monoclonal/46-9333-82

16. Anti-mouse ST2 (IL-33R), PE-Cy7 conjugated, clone RMST2-2, lot 2331972, Invitrogen, Catalog #25-9335-82, Dilution 1:200, https://www.thermofisher.com/antibody/product/IL-33R-ST2-Antibody-clone-RMST2-2-Monoclonal/25-9335-82

17. Anti-mouse Tbet, BV650 conjugated, clone O4-46, lot 1327308, BD Horizon, Catalog #564142, Dilution 1:100, https://www.bdbiosciences.com/en-us/products/reagents/flow-cytometry-reagents/research-reagents/single-color-antibodies-ruo/bv650-mouse-anti-t-bet.564142

18. Anti-mouse Tbet, BV711 conjugated, clone 4B10, lot B268785, BioLegend, Catalog #644819, Dilution 1:100, https://www.biolegend.com/en-us/products/brilliant-violet-711-anti-t-bet-antibody-7952?GroupID=BLG6433

19. Anti-mouse IFN-g, BV605 conjugated, clone XMG1.2, lot B282337, BioLegend, Catalog #505839, Dilution 1:100, https://

	www.biolegend.com/en-gb/products/brilliant-violet-605-anti-mouse-ifn-gamma-antibody-8114<br><br>20. Anti-mouse GATA3, PE conjugated, clone 16E10A23, lot B247091, BioLegend, Catalog #653804, Dilution 1:100, https://www.biolegend.com/en-us/products/pe-anti-gata3-antibody-9076?GroupID=GROUP26<br><br>21. Anti-mouse IL-13, PE-Cy7 conjugated, clone eBio13A, lot 2168366, eBioscience, Catalog #25-7133-82, Dilution 1:100-1:200, https://www.thermofisher.com/antibody/product/IL-13-Antibody-clone-eBio13A-Monoclonal/25-7133-82<br><br>22. Anti-mouse IL-13, PE conjugated 1:100-1:200, clone 16E10A23, lot unknown, BioLegend, Catalog #653804, Dilution 1:100-1:200, https://www.thermofisher.com/antibody/product/IL-13-Antibody-clone-eBio13A-Monoclonal/12-7133-41<br><br>23. BD Pharmingen, AlexaFluor700 Fixable Viability Stain, lot 1153701, Catalog #564997, Dilution 1:1,000-1:5,000, https://www.bdbiosciences.com/en-us/products/reagents/flow-cytometry-reagents/research-reagents/single-color-antibodies-ruo/fixable-viability-stain-700.564997<br><br>24. ThermoScientific, Green Fixable Viability Stain, lot unknown, Catalog #L23101, Dilution 1:1,000, https://www.thermofisher.com/order/catalog/product/L23101<br><br>25. Anti-human Lineage cocktail, Pacific Blue conjugated, clones UCHT1, HCD14, 3G8, HIB19, 2H7, HCD56, lot B323939, Catalog #348805, Dilution 1:50, https://www.biolegend.com/en-us/products/pacific-blue-anti-human-lineage-cocktail-cd3-cd14-cd16-cd19-cd20-cd56-8250<br><br>26. Anti-human Lineage cocktail, eFluor450 conjugated, clones RPA-2.10, OKT3, 61D3, CB16, HIB19, TULY56, HIR2, lot 2331007, Catalog #22-7775-72, Dilution 1:50, https://www.thermofisher.com/antibody/product/Human-Hematopoietic-Lineage-Antibody-clone-RPA-2-10-OKT3-61D3-CB16-HIB19-TULY56-HIR2-Cocktail/22-7775-72<br><br>27. Anti-human CD127 (IL-7Ra), PE conjugated, clone A019D5, lot B322351, Catalog #351304 BioLegend, Dilution 1:200, https://www.biolegend.com/en-us/products/pe-anti-human-cd127-il-7ralpha-antibody-7094?GroupID=BLG9273
Validation	Antibodies 1-22 have been validated by their individual manufacturers for flow cytometric analysis of mouse cells. In house validation confirmed appropriate antigen staining via the use of negative controls prior to use. Reagents 23-24 have been validated by their respective manufacturers to bind to mammalian cells and were tested in erythrocyte-lysed whole blood cells, primary cells, and cell lines. These viability stains were titered in the lab before use. Antibodies 25-27 have been validated by their individual manufacturers for flow cytometric analysis of human cells. In house validation confirmed appropriate antigen staining via the use of negative controls prior to use. For more information on the antibodies used, please visit the links provided above.

Animals and other research organisms

Policy information about studies involving animals; ARRIVE guidelines recommended for reporting animal research, and Sex and Gender in Research

Laboratory animals	C57BL/6, and C57BL/6J × DBA/2 F1 (B6D2) mice were purchased from The Jackson Laboratory, Bar Harbor, ME. The generation of enhanced GFP-expressing C57BL/6 mice has been described previously (PMID: 14715632). Donor and recipient mice were age-matched males between 8 and 16 weeks. Animals were housed on a 12 hour dark/light cycle at 21-22°C, at 30-70% humidity.
Wild animals	No wild animals were used in this study.
Reporting on sex	All murine experiments reported in this study were performed in male mice.
Field-collected samples	No field-collected samples were used in this study.
Ethics oversight	All experiments were performed in accordance with protocols approved by the University of North Carolina Institutional Animal Care and Use Committee (application number 14-001).

Note that full information on the approval of the study protocol must also be provided in the manuscript.

Plants

Seed stocks	No seed stocks were used in this study.
Novel plant genotypes	No novel plant genotypes are described in this study.
Authentication	No plant authentication occurred in this study.

ChIP-seq

Data deposition

☒ Confirm that both raw and final processed data have been deposited in a public database such as GEO.

☒ Confirm that you have deposited or provided access to graph files (e.g. BED files) for the called peaks.

Data access links<br>*May remain private before publication.*	To review GEO accession GSE232003:<br>Go to https://www.ncbi.nlm.nih.gov/geo/query/acc.cgi?acc=GSE232003<br>Enter token kpeliamsltorfof into the box
Files in database submission	blfH3K4ME3_rep1_combined_STARAligned.out.sorted.shiftedExtended.scaled.bw<br>blfH3K4ME3_rep2_combined_STARAligned.out.sorted.shiftedExtended.scaled.bw
Genome browser session<br>(e.g. UCSC)	https://genome.ucsc.edu/cgi-bin/hgTracks?db=mm10&lastVirtModeType=default&lastVirtModeExtraState=&virtModeType=default&virtMode=0&nonVirtPosition=&position=chr2%3A9851697%2D9883981&hgsid=1703848432_VBYmxWFgASZQkhiMShIrXHDLaK8T

Methodology

Replicates	Libraries were prepared from 2 technical replicates and sequenced.
Sequencing depth	Samples were prepared via the Thruplex DNA Seq method and then sequenced on the Illumina HiSeq4000 platform as single-end 1x50 with a single barcode.<br><br>Total number of reads:<br>H3K4me3_rep1: 68,036,253<br>H3K4me3_rep2: 22,742,482<br><br>Uniquely mapped reads:<br>H3K4me3_rep1: 47,271,840<br>H3K4me3_rep2: 13,997,394
Antibodies	Cell Signaling Technologies, anti-H3K4 rabbit mAb, clone C42D8, Lot #10, cat #9751
Peak calling parameters	Read Alignment:<br>STAR (v2.5.2b) with the options: --outFilterScoreMin 1, --outFilterMultimapNmax 1 --outFilterMismatchNmax 2, --chimJunctionOverhangMin 15, --outSAMtype BAM Unsorted, --outFilterType BySJout, --chimSegmentMin 1<br><br>Peaking Calling<br>MACS2 (v.2.1.2, using default parameters).
Data quality	MACS, padj < 0.05 (Default parameters)<br><br>Number of peaks:<br>H3K4me3_rep1: 19,360
Software	star (v. 2.5.2b)<br>samtools (v. 1.3.1)<br>bedtools (v. 2.26)<br>fastqc (v. 0.11.5)<br>fastx_toolkit (v. 0.0.14)<br>cutadapt (v. 1.12)<br>deepTools (v.3.5.2)<br>MACS2 (v.2.1.2)<br>DEseq2 (v.1.40.2)

Flow Cytometry

Plots

Confirm that:

☒ The axis labels state the marker and fluorochrome used (e.g. CD4-FITC).

☒ The axis scales are clearly visible. Include numbers along axes only for bottom left plot of group (a 'group' is an analysis of identical markers).

☒ All plots are contour plots with outliers or pseudocolor plots.

☒ A numerical value for number of cells or percentage (with statistics) is provided.

Methodology

Sample preparation	Animals were euthanized with CO2 and spleen, liver, lung, mesenteric lymph nodes (mLN), and lamina propria (LP) were excised. LP lymphocytes were isolated using the Miltenyi LP dissociation kit (catalog 130-097-410) as per the manufacturer's instructions. Livers and lungs were digested in a solution of 1 mg/ml collagenase A (Roche) and 75 U DNase I (Sigma-Aldrich) in RPMI 1640 with 5% newborn calf serum. Digested tissues were treated with ACK lysis buffer to remove RBCs and were passed through 100 µm cell strainers. Leukocytes were collected at the interface of a 40%:80% Percoll (Sigma-Aldrich)

	gradient in RPMI 1640 with 5% NCS. The pelleted cells were washed in 1x DPBS with 2% FBS. Spleens and mesenteric lymph nodes were teased apart, treated with ACK lysis buffer, and washed in 1 x DPBS with 2% FBS. Single cell suspensions were stained with surface antibodies, washed, and then treated with a Fixable Viability Stain. For intracellular cytokine or transcription factor staining, cells were fixed and permeabilized with the BD Fix/Perm or eBioscience FoxP3 TF kits, respectively, prior to incubation with intracellular or intranuclear antibodies.<br><br>For sorting experiments: Single cell suspensions were stained with an e450 Lineage antibody cocktail (Invitrogen, 88-7772-72) and BD Horizon AlexaFluor 700 Fixable Viability Stain (BD 564997). Cells were sorted based on GFP expression and GFP+ cells were collected into cR10 prior to downstream processing.
Instrument	Analysis: BD LSRII, BD LSRFortessa (BD Bioscience) or MACS Quant 16 (Miltenyi). Sorting: BD FACSAria II (BD Bioscience).
Software	Flow cytometry data were collected with BD FACSDiva software when using a BD analyzer, or with the MACSQuantify software when using the MACSQuant 16 analyzer. All .fcs files were analyzed with FlowJo (Version 10.8).
Cell population abundance	A minimum of approximately 10,000 cells were analyzed for any given cytometry sample. Following ex vivo expansion, cell cultures contained 85%+ live ILC2s in both the mouse and human setting. For experiments were FACS sorting was utilized, live, lineage-, GFP+ cells generally represented between 0.5-1% of total cells.
Gating strategy	ILC2s were defined as live (fixable viability stain negative) singlets negative for staining with the Lineage antibody cocktail and positive for ST2/IL-33R (mouse) or CD127 (human). Upon gating for single, viable ILC2s, fluorescence minus one (FMO) or controls were used to establish negative and positive gates. In the case of cells that underwent PMA and ionomycin stimulation for intracellular cytokine or transcription factor staining, unstimulated cells (treated with Brefeldin A alone) were reserved as a negative control. All experiments included unstained and single color compensation controls.

☒ Tick this box to confirm that a figure exemplifying the gating strategy is provided in the Supplementary Information.
